# Single-Fraction SBRT for Locally Advanced Pancreatic Cancer Using Total Intravenous Anaesthesia and Optical Surface Guidance: Technique and Preliminary Results

**DOI:** 10.3390/cancers17193093

**Published:** 2025-09-23

**Authors:** Hrvoje Kaučić, Maja Karaman Ilić, Domagoj Kosmina, Ana Mišir Krpan, Sunčana Divošević, Asmir Avdičević, Hrvoje Feljan, Matea Lekić, Karla Schwarz, Dragan Schwarz

**Affiliations:** 1 Specialty Hospital Radiochirurgia Zagreb, 10431 Sveta Nedelja, Croatiadragan.schwarz@radiochirurgia.hr (D.S.); 2 Medical Faculty Osijek, University Josip Juraj Strossmayer in Osijek, 31000 Osijek, Croatia; 3 Faculty of Dental Medicine and Health Josip Juraj Strossmayer, University of Osijek, 31000 Osijek, Croatia; 4 Department of Physics, Faculty of Science, University of Zagreb, 10000 Zagreb, Croatia; 5 Medical Faculty Mostar, University of Mostar, 88000 Mostar, Bosnia and Herzegovina; 6 School of Medicine, University of Zagreb, 10000 Zagreb, Croatia; 7 Medical Faculty Pula, Juraj Dobrila University of Pula, 52100 Pula, Croatia

**Keywords:** locally advanced pancreatic cancer, stereotactic ablative radiotherapy, stereotactic body radiotherapy, total intravenous anaesthesia, tumour motion management

## Abstract

Background: The aim of this retrospective, single-arm study was to present the technique and preliminary efficacy and safety results of a single-fraction SBRT for LAPC using total intravenous anaesthesia and optical surface guidance as motion management. Methods: Fifty-five patients with locally advanced pancreatic cancer were treated with SBRT with a single-fraction receiving a median BED_10_ = 128.9 Gy. Forty-two patients received systemic treatment. End points were OS, FFLP, PFS, and toxicity. Actuarial survival analysis and univariate analysis were investigated. Results: The median follow-up was 15 months, mean OS was 18 months (95% CI: 16.7 to 19.3), and the one-year FFLP and 1-year OS were 100% and 90.9% (95% CI: ± 1.5%), respectively. Median PFS was 12 months (95% CI: 9.5 to 14.4), and 1-year PFS was 85.5% (95% CI: ± 1.4%). Thirty-five patients (63.6%) were alive at the time of analysis. No acute/late toxicity > G2/G1 was reported. Conclusions: SBRT for LAPC using total intravenous anaesthesia and optical surface guidance presented as an effective and safe treatment with very low toxicity.

## Simple Summary

Unresectable, locally advanced pancreatic cancer represents a particular problem for SBRT due to the tumour and surrounding healthy tissue, respiratory, and other movements, limiting the tumour dose. The aim of this study was to investigate the efficacy and safety of single-fraction SBRT for LAPC with dose escalation using total intravenous anaesthesia and optical surface guidance as motion management. With this technique, we aimed to almost completely mitigate the movements of the tumour and OARs, imitating the non-moving target SBRT, and this is the first reported application of this approach for LAPC. Using very narrow safety margins around the lesion during the treatment, we were able to effectively spare surrounding healthy organs and safely apply a median biological effective dose ≥ 128.9 Gy. Especially when combined with systemic therapy, the treatment resulted in very favourable one-year local tumour control of 100% and a median overall survival of 18 months, with no grade > 2 toxicities.

## 1. Introduction

Pancreatic cancer is among the leading causes of cancer-related deaths around the world, with the 5-year survival rate generally less than 10% [1,2]. Only a small portion of patients (<20%) are candidates for radical surgery with an R0 margin that can significantly prolong survival, as the tumour is typically diagnosed at an advanced stage. An estimated 30–40% of patients with pancreatic cancer present with a locally advanced stage at the time of diagnosis [3].

Successful stereotactic body radiotherapy (SBRT, also called stereotactic ablative radiotherapy, SABR) of pancreatic lesions leads to better local control (LC), but requires the application of very high biologically effective doses (BED) (≥100 Gy), and a significant positive impact of LC on overall survival (OS) has been shown [4,5,6]. The tumour lesion and surrounding healthy tissues are subject to significant physiological movements, with the vast majority of the movements caused by respiration, and this is most evident in regions close to the diaphragm—the lung bases and upper abdomen [7,8]. Monitoring tumour respiratory movements in free breathing (FB) using 4D CT and compensating them with an expanded internal tumour volume (ITV) is a widely used approach, but it can limit the possibilities of applying the above-mentioned ablative doses, reducing the effectiveness of SBRT [9,10,11,12]. Respiratory mitigation using abdominal compression, respiratory gating, and fiducial-based intrafractional motion tracking (typically with robotic arm-based linacs) are also used as motion management for patients in FB. Voluntary breath hold (BH) in the inhale or exhale phase significantly reduces respiratory movements and is most often used with surface guidance or intrafractional tracking with cine magnetic resonance imaging (MRI). It is very effective and favourable in terms of dose distribution, but requires strict patient cooperation, is not completely reproducible, and significantly prolongs the duration of SBRT [9,13,14].

At the Specialty Hospital Radiochirurgia Zagreb, from April 2017 to September 2022, we used the Calypso Tracking System (Varian Medical Systems) for intrafractional fiducial-based tumour motion tracking for upper-abdominal tumours, mainly for locally advanced pancreatic cancer (LAPC) [15]. We considered Calypso (when applicable) as probably the best intrafractional motion management during SBRT [16]. However, in 2022, Varian Medical Systems [17] announced that they would no longer support the Calypso tracking system from 2024 onward and would not be producing new fiducials, so we had to adapt our methodology to the phase out of technology.

We came up with a new approach—SBRT for upper-abdominal lesions (including LAPC) in total intravenous anaesthesia (TIVA) with assist-control mechanical ventilation (ACMV) combined with the AlignRT (Vision RT, London, UK) optical surface guidance (OSG) system—for intrafractional motion management. Using TIVA with ACMV to control the patients’ BH (named as “TIVA controlled BH”), respiratory movements were almost completely eliminated, and bowel movements were widely reduced. Consequently, the tumour target became almost motionless. Under these conditions, the clinical target volume-planning target volume (CTV-PTV) margins were reduced to 2 mm, thus enabling high precision and safe ablative dose delivery to the tumour, with excellent protection of the surrounding organs at risk (OAR). We started performing SBRT in TIVA controlled BH in October 2022. Using this technique, we managed to apply prescribed doses of up to 35 Gy (BED10 = 157.5 Gy) in a single-fraction to the pancreatic tumour.

This paper intends to present TIVA controlled BH with OSG as an intrafractional motion management technique during single-fraction SBRT for LAPC as well as the preliminary clinical results (LC, OS and toxicity) with the aim to show at least the non-inferiority of this approach compared with Calypso. To our best knowledge, there have been no published data on the usage of TIVA with ACMV combined with OSG as an intrafractional motion management system during SBRT for LAPC, although some experiences on using total endotracheal anaesthesia during SBRT have been reported. This motion management technique during SBRT can be used for other upper-abdominal lesions.

## 2. Materials and Methods

### 2.1. Patients

The medical data of 55 patients diagnosed with LAPC, treated between December/2022 and June/2024 and regularly followed up at our institution, were analysed and enrolled in this retrospective, single-arm, and single-institution experimental study, which was approved by the institutional Ethics Committee. The patients’ characteristics are shown in Table 1.

All medical procedures in our study were in accordance with the 1964 Helsinki Declaration and its later amendments and met all comparable international and national ethical standards. All patients involved in the study signed their informed consent. Our institution’s multidisciplinary team (MDT), consisting of radiation oncologists, pancreatic/biliary surgeons, radiologists, medical physicists, and medical oncologists, discussed and approved all patients for SBRT. Inclusion criteria were unresectable, histologically proven pancreatic adenocarcinoma; ECOG 0–2; age ≥ 18; no signs of regional or distant metastasis and gastric or duodenal obstruction on diagnostic imaging; and no previous abdominal radiotherapy.

The recommendations from the National Comprehensive Cancer Network (NCCN) guidelines and American Hepato–Pancreato–Biliary Association/Society of Surgical Oncology/Society for Surgery of the Alimentary Tract were followed to define unresectable pancreatic cancer [6,18]. The multi-slice computed tomography (MSCT) pancreas protocol was performed for each patient, and 3T MRI of the abdomen and/or positron emission tomography/computed tomography (PET/CT) were added as needed. Each patient was examined by our anaesthesiologist and radiation oncologist for final approval.

Forty-two patients (76.4%) received systemic treatment. Thirty-five patients (83.3%) started the systemic treatment after SBRT, and seven patients (16.7%) started the systemic treatment (2 cycles) before SBRT and continued after SBRT. To avoid possible toxicities of concurrent application, systemic therapy was held at least one week before and after SBRT.

### 2.2. Patients’ Preparations for the Treatment

We provided all patients with written recommendations on diet and medications—proton pump inhibitors and antiflatulent drugs—to reduce flatulence and weight loss, in order to minimize unwanted daily anatomical variations. The time from planning to the start of treatment was 7–10 days.

### 2.3. Total Intravenous Anaesthesia with Assist-Control Mechanical Ventilation

The anaesthesiologic assessment included a clinical examination of the patient, a review of the patient’s medical documentation with special attention to comorbidities, and a review of the patient’s laboratory findings. Patients with a history of heart and/or lung disease had to have a recent examination by a cardiologist (heart ultrasound included) and/or a pulmonologist (spirometry included).

Absolute contraindications for TIVA with ACMV were recent myocardial infarction; ejection fraction below 40%; cardiac failure; uncontrolled hypertension and cardiac rhythm disturbance with haemodynamic repercussions; severe pulmonary hypertension; pulmonary fibrosis; severe obstructive-restrictive ventilation disorders; and pleural effusion. All patients were ASA II or ASA III according to the American Society of Anaesthesiology (ASA) classification.

Patient preparation before anaesthesia for planning and SBRT was the same for all patients. The patients were hospitalised on the day of planning and treatment. If additional treatment (e.g., monitoring, correction and optimisation of vital functions, volume replacement, and achieving euglycaemia and electrolyte balance) was needed, the patients were hospitalised the day before.

Thirty minutes after preparation and premedication (i.e., gastroprotection and sedation with pantoprazole, metoclopramide, and midazolam per os), patients were transferred to the operating room and placed on TIVA. The procedure started with patient preoxygenation. The medications used for the induction and maintenance of anaesthesia were midazolam and propofol for sedation and hypnosis, sufentanyl for analgesia, and rocurronium for muscle relaxation, in the prescribed dose per kilogram of body weight.

A supraglottic device I-Gel (Intersurgical Ltd. Wokingham, Berkshire, UK) was placed to establish the airway [19]. In cases of inability to place the I-Gel or excessive “air leakage”, the patient was intubated.

Patients were ventilated with a volume control ventilation-tidal volume of 6 mL/kg body weight. The selected ventilation modality was applied for both planning and SBRT.

Intraprocedural monitoring consisted of electrocardiogram, non-invasive blood pressure (NIPB), oxygenation saturation (SpO_2_), and end-tidal CO_2_ monitoring. During SBRT, NIBP monitoring was substituted with invasive arterial pressure (IBP) monitoring. The duration of the SBRT procedure makes NIBP unsuitable, as NIBP does not have the temporal resolution that IBP has. The need for the accurate monitoring of the effects of respiratory manoeuvres on the haemodynamics justified the invasiveness of the IBP placement.

After the induction of anaesthesia and airway clearance, the patients were transferred to the MSCT device for planning or to linac for SBRT treatment as needed. At the time of planning or SBRT, the selected controlled ventilation modality was terminated using an “expiratory” BH for 30 s on average, alternating with preset ventilation. The manoeuvre was repeated as necessary. Blood gasses were taken during the SBRT procedure, after induction, and immediately after the procedure was completed. This primarily served to monitor the changes in the partial pressure of carbon dioxide in arterial blood caused by ventilation interruptions.

Upon completion of the procedure (planning or SBRT), awake, spontaneously breathing, and haemodynamically stable patients were brought back to the department for continued recovery with the monitoring of vital functions (heart rate, NIBP, SpO_2_). After an average two-hour stay in the department, the patients were discharged home.

### 2.4. Optical Surface Guidance

The AlignRT (Vision RT, London, UK) monitoring system was used for OSG, combined with cone beam CT (CBCT) for image guidance (IG) [20]. Two systems were used for both patient positioning and motion tracking. According to the manufacturer’s specifications, the AlignRT system tracks any movements of the body contour in real-time with a frequency of 5–10 Hz, a lag time < 100 milliseconds, and submillimetre accuracy.

The patient’s body contour on planning MSCT was defined as the default body contour (DBC), and the region of interest (ROI) was specified as a segment of the patient’s skin above the PTV. Anatomical position of the tumour on the planning MSCT was the therapeutic position.

AlignRT was used for “on-off” gating management during the treatment and monitoring the alignment of the ROI of the DBC with the patient’s body contour ROI on the treatment table. Gating windows allowed for a tolerable mismatch of ROIs in any direction within 2 mm. As long as the ROIs were aligned within the stated gating windows, the beam was “on”. Whenever the ROIs’ mismatch exceeded the gating windows, AlignRT automatically shot the beam off.

### 2.5. Tumour Movements During SBRT in TIVA Controlled BH-Assessed by Calypso System

**Figure 1:**
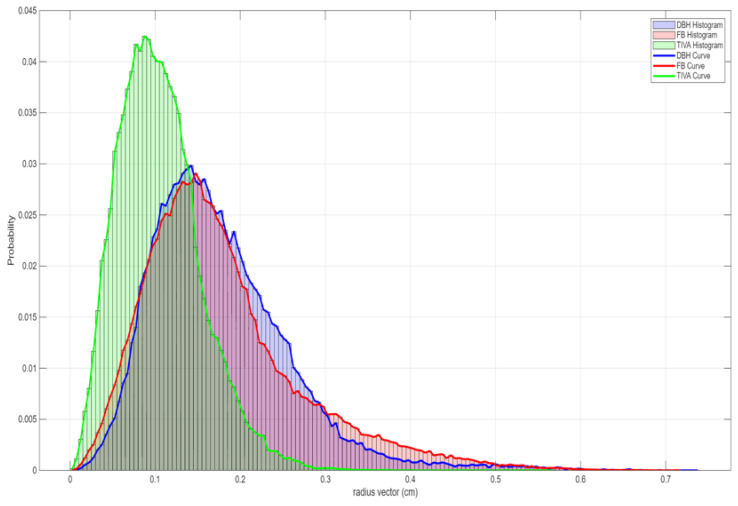
Comparative display of the radial movement histograms and curves for patients treated in TIVA controlled BH (TIVA); patients treated in voluntary BH (DBH); and patients treated in FB (FB).

During 2023, we continued with the implantation of Calypso fiducials in upper-abdominal lesions (UALs) and treated those patients with SBRT in TIVA controlled BH, but using the Calypso system as motion management. There was a total of 23 patients with UALs that were treated in such manner. We compared this subgroup to 23 patients with UALs treated in voluntary BH with implanted Calypso fiducials and 23 patients with UALs treated in FB with implanted Calypso fiducials. By analysing radial movement histograms (Figure 1) of the initial positioning displacement and treatment length, we came to the following conclusions:

- Movement analysis and positioning accuracy: TIVA controlled BH achieved the smallest geometric displacement (0.05 cm) and rotation (3.8 degrees), indicating improved positioning stability.
- Movement tracking and treatment time: TIVA controlled BH reduced the median radial movement during treatment to 0.26 cm with a total treatment time of 46.2 min (~2 h) compared with FB and voluntary BH, which had higher movement and longer treatment times. During beam-on, TIVA controlled BH showed the lowest median radial movement (0.1 cm) and the longest average treatment interval (19.5 s), indicating better motion control
- Movement histograms and data visualisation: Normalised histograms of tracking movement during all treatment phases and beam-on periods confirmed reduced motion with TIVA controlled BH compared with FB and voluntary BH, supporting improved treatment precision.
- Clinical implications and future directions: TIVA controlled BH reduced uncertainties and improved repeatability and imaging, enabled single-fraction treatment with elective lymph node irradiation, and eliminated the need for fiducials.

### 2.6. Stereotactic Ablative Radiotherapy

The TIVA procedure was essentially used to perform the most identical exhale BHs for the patients during the planning and SBRT procedures by controlling respiratory shifts.

A contrast-free MSCT scan in TIVA controlled BH with a slice thickness of 1 mm was performed for planning purposes. Additionally, contrast-enhanced MSCT (with late arterial phase) and contrast-free MRI of the upper abdomen (T1 and T2 with high spatial fidelity) in voluntary BH were acquired for all patients. The contrast enhanced MR sequences tended to overestimate the actual volumes, so the contrast-free MR imaging was used.

The planning MSCT was co-registered (with deformable registration methods as needed) with MRI and contrast-enhanced MSCT. The CTV was defined as the gross tumour volume (GTV), with no additional margins and contoured on the T1 or T2 images of the MRI. Further corrections of CTV on planning MSCT scans were performed if necessary. We followed the European Society for Radiotherapy and Oncology (ESTRO), American Society for Radiation Oncology (ASTRO), and NCCN recommendations on CTV and OAR delineation.

**Figure 2:**
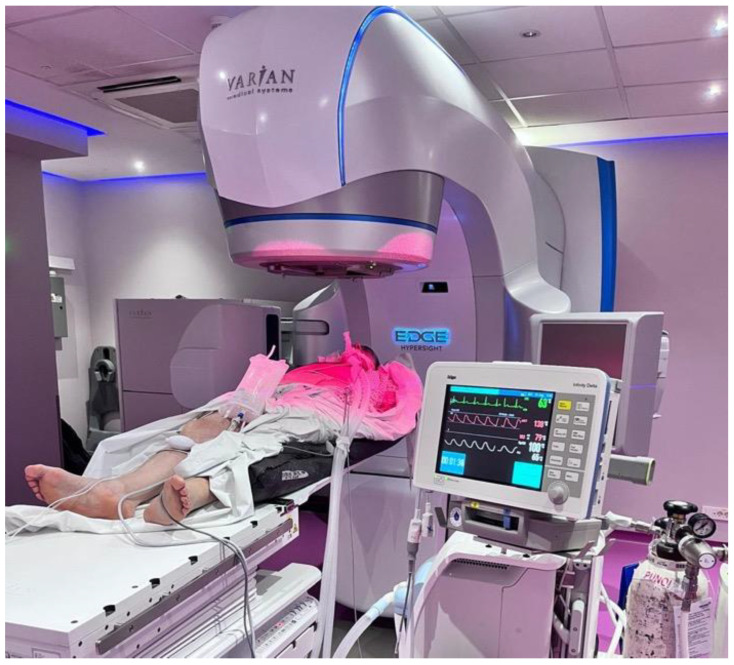
Patient’s position on a treatment table and anaesthesiologic monitoring equipment during SBRT in TIVA controlled BH and OSG.

Patients were in a supine position with their arms by their side, on a vacuum pillow (Figure 2). No additional immobilisation methods were used. A Varian EDGE^®^ linear accelerator (Varian Medical Systems, Palo Alto, CA, USA) was used for treatment delivery.

All SBRT plans were optimised and delivered using Volumetric Modulated Arc Therapy (VMAT); 6–8 partial arcs per treatment on average, with no treatment table rotation. Flattening filter free (FFF) 10 MV photon beams and dose rates up to 2400 MU/min were used. Criteria for the stated beam energy and dose delivery technique were: (1) to achieve the best dose distributions; and (2) to provide plans with low modulation and high QA passing rates. Alpha/beta ratio = 10 Gy was used to calculate the biological effective dose to the tumour (BED10).

The SBRT procedure was performed in a single-fraction manner using two PTVs for regional lymph nodes (as the NCCN Guidelines suggest that broad coverage of mesenteric vasculature ± nodal regions should be considered when feasible) and for the primary tumour lesion.

Regional lymph nodes received the elective dose of 15 Gy (BED10 = 37.5 Gy) to the PTV borders, with a mean dose of 18 Gy inside the PTV.

Pancreatic tumours received the simultaneous integrated boost (SIB) with the prescribed dose of 30–35 Gy (BED10 = 120–157.5 Gy) in the following manner:

- The mean dose to the SIB-PTV was considerably higher than the prescription dose;
- No planning constraints on the dose maximums were set as long as they were inside the SIB-PTV;
- The optimisation of the dose distribution was performed with the purpose of achieving a required target coverage of V (98–99.5%) = 80% of the prescribed dose for the SIB-PTV.

The result was a highly heterogeneous dose distribution inside the SIB-PTV. The average maximum was 138.7% (ranging 126.8 to 148.4%) of the prescription dose.

The PTV-CTV margin was defined according to van Herk’s formula: 2.5Σ + 0.7σ → 2.5 × 0.4 mm + 0.7 × 1.4 mm = 2 mm, in the X, Y, and Z directions:

- Σ = 0.4 mm, as determined by end-to-end tests; for systemic error;
- σ = 2 mm, defined as gating windows; for random error.

Consequently, both PTVs were generated using a 2 mm margin to CTVs. The average conformity index (the ratio of the volume of the 80% isodose line to the volume of the SIB-PTV) was 1.07 (ranging 1.03 to 1.12).

The OARs were divided into two groups:

1. Primary (directly adjacent to pancreas and highly radiosensitive) OARs: stomach, duodenum, small bowel;
2. Other OARs: liver, great vessels, spinal cord, kidneys.

We followed dose–volume constraints according to Murphy et al. for the primary OARs, and the AAMP recommendations for the other OARs, respectively [21,22]. As long as the stated OAR constraints were met, the target coverage was prioritised over OAR sparing. Table 2 summarises the dose–volume constraints.

Initial positioning was performed using AlignRT and co-registration of the planning MSCT with CBCT using soft tissue and bony anatomy. After the treatment started, the beam was on as long as the patient was both in TIVA controlled BH and the body contour was within the gating windows of AlignRT.

CBCT was regularly repeated after 50% of the dose was delivered to recheck any possible mismatch of CTVs or OARs, and if needed, further corrections were made using CBCT. If AlignRT detected significant and permanent deviation of the ROIs at any time during the treatment, the procedure was repeated.

According to the system’s reports, the “beam on” was on average 11.6% of the treatment time, calculated from the first beam engaged to the end of the treatment. The average treatment time was 46 min, while the TIVA procedure took up an additional 68 min on average.

### 2.7. Response Evaluation and Follow-Up

Follow-up was regularly scheduled every 3 months after SBRT by the treating radiation oncologist with clinical examination, a contrast-enhanced MSCT scan, and blood tests including Ca 19-9. Contrast-enhanced MRI imaging was added in the case of regional and/or distant relapse suspicion. RECIST criteria were used for the analysis of local and distant control [23]. The National Cancer Institute (NCI) Common Terminology Criteria for Adverse Events (CTCAE) v 5.0 general guidelines were used to score acute and late toxicity.

### 2.8. Statistical Analysis

End points were OS, LC (i.e., freedom from local progression-FFLP), progression-free survival (PFS), and toxicity. One-year OS was calculated as a ratio of patients that survived at least 12 months and all patients. FFLP was defined as radiological progression of the primary lesion within the PTV. FFLP and PFS were calculated from the time of diagnosis to the first radiological assessment of local progression or regional/distal progression, respectively. Patients that did not develop disease progression were censored at the date of the last scan. OS, FFLP, PFS, and rates were calculated from the time of diagnosis to death following the Kaplan–Meier method. The log-rank test statistic was used for univariate analysis, with significant difference considered when *p* ≤ 0.05.

## 3. Results

**Figure 3:**
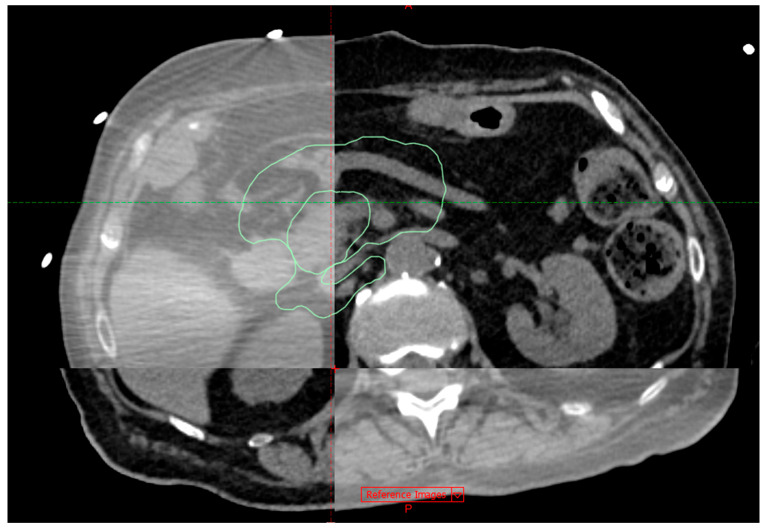
Planning MSCT co-registration with CBCT on a treatment table with the PTV conture (green) displayed.

All patients completed the treatment in a single-fraction with no delays. Median follow-up was 15 months (range 7–32 months). Forty-one patients (74.5%) received a prescribed dose of 31.25 Gy, which was a median dose. Ten patients (18.2%) received a prescribed dose of 30 Gy—the dose was reduced to meet the OAR constraints. Four patients (7.3%) with particularly favourable anatomy received a prescribed dose of 35 Gy. Treatments lasted between 30 and 70 min, and there were 10–14 beam-ons per treatment, each lasting approximately 18–36 s, and the times between them were beam-offs. Eleven patients (20%) had Calypso fiducials implanted. Figure 3 represents a planning MSCT co-registration with CBCT on a treatment table.

**Figure 4:**
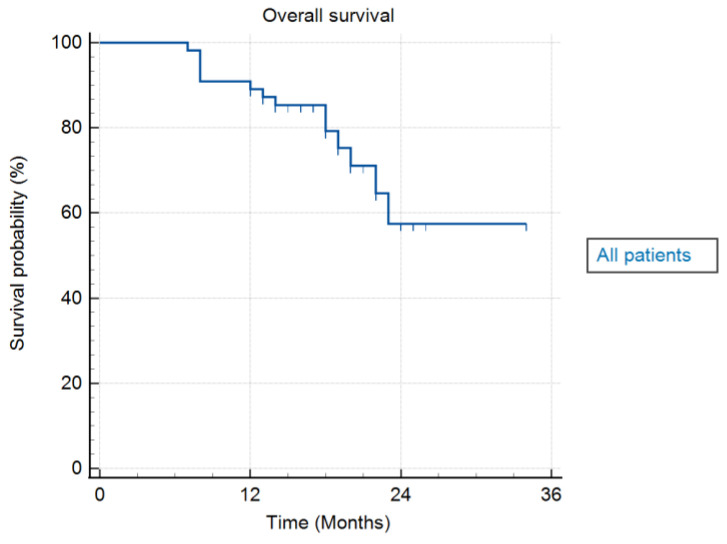
Actuarial curve of overall survival for all patients.

Median OS was 18 months (range 7–34 months). On a univariate survival analysis using Kaplan–Meier curves, the median OS was not achieved, as > 50% of patients were alive at the time of the analysis. Median time from diagnosis to SBRT was 2 months (range 1–5 months). One-year FFLP, one-year OS, and one-year PFS were 100%, 90.9%, and 85.5%, respectively. Table 3 summarises the results for FFLP, PFS, and OS. Figure 4 shows an actuarial curve for OS.

**Figure 5:**
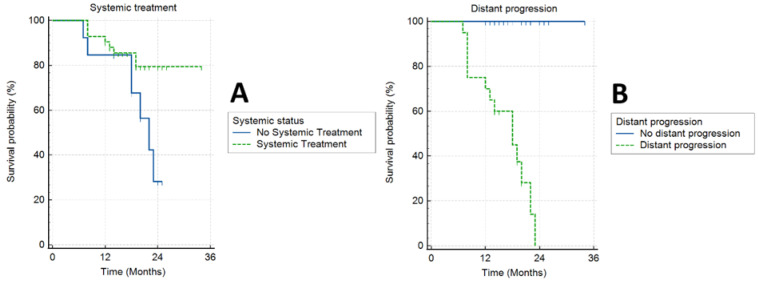
Actuarial curves of survival for patients receiving/not receiving systemic treatment (**A**) and patients with/without distant progression (**B**).

Forty-two patients (76.4%) received systemic treatment. In the actuarial analysis, patients that received chemotherapy had a significantly better OS (log-rank, *p* = 0.05) (Figure 5A). One patient (2%) had radiological local disease progression (at 23 months, accompanied by distal progression). Other patients had either local regression or stable local disease. Seven patients (12.7%) had complete local response to treatment, with no visible primary lesion on diagnostic imaging during follow-up, typically 12 months after SBRT.

Median PFS was 12 months (range 7–23 months). Twenty patients (36.4%) had distant progression of the disease, and 35 patients (63.6%) had systemic stable disease. In the actuarial analysis, patients that had no distant progression had a better OS (log-rank, *p* = 0.0001) (Figure 5B).

Median tumour volume (GTV) was 32.6 cm^3^ (range 6.7–98.1 cm^3^). In the actuarial analysis, there was no impact of tumour volume on OS.

Acute and late toxicities were assessed according to the NCI/CTACE v 5.0 general guidelines. Acute toxicities grade 1 (defined as mild symptoms) and grade 2 (defined as moderate symptoms), for example, fatigue, nausea, abdominal spasm, or abdominal pain were successfully treated and resolved with proton pump inhibitors, antiemetics, and spasmolytics. Late toxicities grade 1 (e.g., abdominal spasm or pain, and/or gastroesophageal reflux, developing six months or later after SBRT) were successfully treated and resolved with similar symptomatic treatment. No patients reported grade 3 (defined as severe or medically important symptoms) and grade 4 (defined as life threatening and/or urgent symptoms) acute toxicities. Additionally, no patient reported grade 2 late toxicities. The toxicity summary is shown in Table 4.

Forty-one patients (74.5%) were alive at the time of analysis. Thirty-five patients (85.4%) from this subgroup received systemic treatment.

## 4. Discussion

SBRT, as the treatment for patients with upper abdominal (including pancreatic) lesions, is today considered a well-established and effective treatment. Positive impacts of dose escalation to LC and OS have also been shown.

Single-fraction SBRT is very appealing, although it is still not a thoroughly investigated concept in radiation oncology [24,25]. An avoidance of single-fraction SBRT for pancreatic lesions (and upper abdominal targets, in general) still exists in general, primarily due to increased gastrointestinal (GI) toxicities previously reported [26,27]. Furthermore, multifractional SBRT regimes (3–5 fractions) became generally established to the present day as they have been shown to limit GI toxicity [28].

Motion management techniques in FB essentially have two approaches: (1) Establish the whole volume of lesion’s movements and irradiate that volume (e.g., 4D CT generated ITV); and (2) track the movements in real-time and irradiate the moving target (e.g., fiducial based), or to irradiate the lesion in a certain anatomical position (e.g., respiratory gating). On the other hand, techniques in BH attempt to mitigate or cease the lesion’s movements and reduce the PTV-CTV margins (e.g., voluntary BH). In both cases (FB and BH), there are still considerable uncertainties that limit considerable dose escalations and single-fraction SBRT in the upper-abdominal region.

The motion management technique presented in our paper essentially has the opposite approach and was actually inspired by intracranial stereotactic radiosurgery (SRS)—not to track the movements, but to irradiate the non-moving target. We aimed to minimise the movements of the tumour and OARs in a way as to “mimic” the SRS. The encouraging reports from Greco et al. on prostatic cancer single-fraction SBRT were our further inspiration [29,30].

Eleven patients (20%) were treated with implanted Calypso fiducials, and our calculations showed that the lesions’ radial movements were < 1 mm. Guided by van Herk’s formula, we set both the PTV-CTV margin and AlignRT’s gating windows to 2 mm. Consequently, a superior OARs sparing was achieved, which enabled safe single-fraction dose escalation to the tumour with extremely heterogeneous dose distributions that might have been potentially beneficial in treating central, hypoxic radioresistant areas.

Although we could not find any published data on the use of TIVA with ACMV combined with OSG as an integrated intrafractional motion management technique during SBRT for LAPC, recently, non-invasive mechanical ventilation has been explored in radiotherapy [31,32,33,34,35,36]. Additionally, several forms of high frequency ventilation with or without anaesthesia have been applied in radiotherapy to suppress respiratory motion in the thorax and upper abdomen [37,38,39,40,41]. Mechanical ventilation provides two ways to reduce organ movement: by regulating breathing [35,36] and by prolonged BH combined with preoxygenation and induced hypocapnia [32,33,42]. There are presumed advantages of TIVA with ACMV—no respiratory training or preparation for the patient is required; no hypercapnia during the procedure; the entire procedure takes less time compared with SBRT in voluntary BH; and each respiratory cycle and the ventilation parameters are identical during planning and SBRT. Possible disadvantages are that the patient needs to be put under TIVA twice, and the patient is dislocated from the anaesthesiologic team during the procedure, which makes monitoring more challenging.

The considerable proportion of patients in our study (74.5%) were alive at the time of the analysis, and all patients in this subgroup were free from local and distant relapse. Furthermore, 98% of all patients had local regression or stable disease, with 12.7% patients having complete local response to treatment, with no visible primary lesion on diagnostic imaging, which could lead to the potential conclusion that a dose escalated single-fraction SBRT approach could yield better clinical outcomes.

The toxicity profile of SBRT in our study was very acceptable, as patients reported grade ≤ 2 acute and grade ≤ 1 late toxicity, but due to the relatively short follow-up, only a minor part of late toxicity developing after 12 months was captured. It is our impression that the general absence of severe toxicity in our study could have been related to successful OARs sparing.

Possible disadvantages and biases of this study arise from its retrospective and single institution nature, short median follow-up, and the fact that only patients eligible for TIVA were selected and included. The authors are also aware that TIVA has potential dangers for patients, but at the same time, allows for safe single-fractioned and dose escalated SBRT, resulting in possible improvements in LS and OS. In our opinion, the presented preliminary results could be a potentially valuable and encouraging basis for future prospective studies on the role of TIVA during SBRT for upper-abdominal tumours in comparison to standard procedures.

## 5. Conclusions

The TIVA controlled BH combined with OSG as an intrafractional motion management system during dose escalated single-fraction SBRT for LAPC presented in our study is an effective and safe local treatment for LAPC. Satisfactory LC and OS with very acceptable toxicities were shown. Our results indicate that the TIVA controlled BH and OSG enabled effective OARs sparing with a high dose heterogeneity inside the lesion and steep dose falloff outside, consequently allowing for safe dose escalated single-fraction SBRT. This approach could lead to possible improvements in the clinical outcomes for patients and could be considered as part of the multimodality treatment for LAPC.

Future prospective clinical trials are needed to define the role of dose escalated single-fraction SBRT in the improvement of clinical outcomes for patients with LAPC.

## Figures and Tables

**Table 1:** Patients’ characteristics.

Number of patients	55
Mean age in years (range)	69 (42–86)
Sex (M:F)	31:24
ECOG PS	
0	12 (21.8%)
1	38 (69.1%)
2	5 (9%)
Primary site	
Head	43 (78%)
Body/tail	12 (22%)
Systemic treatment	
FOLFIRINOX	23 (42%)
Gemcitabine-based	19 (35%)
No systemic treatment	13 (24%)
Median gross tumour volume	32.6 cm^3^ (range 6.7–98.1 cm^3^)

Abbreviations: ECOG PS-Eastern Cooperative Oncology Group Performance Status.

**Table 2:** Dose–volume constraints.

Primary OAR (Stomach, Duodenum, Small Bowel)	Dmax (0.03 cm^3^) < 23 Gy V(20 Gy) < 3.3 cm^3^ V(15 Gy) < 9.1 cm^3^
Liver	V(9.1 Gy) < 700 cm^3^
Great vessels	Dmax < 37 Gy
Spinal cord	Dmax < 14 Gy
Kidneys	V(8.4 Gy) < 200 cm^3^

**Table 3:** Summary of the results for FFLP, PFS, and OS.

End Points	Median	1 Year
FFLP	23 months *	100% *
PFS	12 months (95% CI: 9.6 to 14.4)	85.5% (95% CI: ±1.4%)
OS	18 months (95% CI: 16.7–19.3)	90.9% (95% CI: ±1.5%)

* One case of local failure at 23 months. Abbreviations: CI, confidence interval; FFLP, freedom from local progression; OS, overall survival; PFS, progression-free survival.

**Table 4:** Toxicity.

NCI/CTACE v 5.0	Acute (Patients, %)	Late (Patients, %)
Grade 1	35 (63.6%)	4 (7.3%)
Grade 2	9 (16.4%)	none

## Data Availability

Research data are stored in an institutional repository and will be shared upon request to the corresponding author.

## Abbreviations

SBRT	Stereotactic body radiotherapy
SABR	Stereotactic ablative radiotherapy
LC	Local control
BED	Biologically effective dose
OS	Overall survival
FB	Free breathing
ITV	Internal tumour volume
BH	Breath hold
CTV	Clinical target volume
PTV	Planning target volume
MRI	Magnetic resonance imaging
LAPC	Locally advanced pancreatic cancer
TIVA	Total intravenous anaesthesia
ACMV	Assist-control mechanical ventilation
OSG	Optical surface guidance
OAR	Organs at risk
ECOG PS	Eastern Cooperative Oncology Group Performance Status
MDT	Multidisciplinary team
NCCN	National Comprehensive Cancer Network
MSCT	Multi-slice computed tomography
PET/CT	Positron emission tomography/computed tomography
ASA	American Society of Anaesthesiology
NIPB	Non-invasive blood pressure
IBP	Invasive arterial pressure
CBCT	Cone beam CT
IG	Image guidance
DBC	Default body contour
ROI	Region of interest
GTV	Gross tumour volume
ESTRO	European Society for Radiotherapy and Oncology
ASTRO	American Society for Radiation Oncology
SIB	Simultaneous integrated boost
NCI	National Cancer Institute
CTCAE	Common Terminology Criteria for Adverse Events
FFLP	Freedom from local progression
PFS	Progression-free survival
CI	Confidence interval
GI	Gastrointestinal
